# Effects of dietary addition of ellagic acid on rumen metabolism, nutrient apparent digestibility, and growth performance in Kazakh sheep

**DOI:** 10.3389/fvets.2024.1334026

**Published:** 2024-02-06

**Authors:** Wenjie Zhang, Feier Ren, Changjiang Zang, Fan Yang, Xuanyue Li, Xinxin Huang, Kaixu Chen, Xiaobin Li

**Affiliations:** College of Animal Science and Technology, Xinjiang Key Laboratory of Meat & Milk Production Herbivore Nutrition, Xinjiang Agricultural University, Urumqi, China

**Keywords:** growth performance, apparent nutrient digestibility, ellagic acid, Kazakh sheep, rumen fermentation, bacterial diversity, antioxidant capacity

## Abstract

Plant extracts have shown promise as natural feed additives to improve animal health and growth. Ellagic acid (EA), widely present in various plant tissues, offers diverse biological benefits. However, limited research has explored its effects on ruminants. This study aimed to investigate the effects of dietary addition EA on rumen metabolism, apparent digestibility of nutrients, and growth performance in Kazakh sheep. Ten 5-month-old Kazakh sheep with similar body weight (BW), fitted with rumen fistulas, were randomly assigned to two groups: the CON group (basal diet) and the EA group (basal diet + 30 mg/kg BW EA). The experiment lasted 30 days, and individual growth performance was assessed under identical feeding and management conditions. During the experimental period, rumen fluid, fecal, and blood samples were collected for analysis. The results indicated a trend toward increased average daily gain in the EA group compared to the CON group (*p* = 0.094). Compared with the CON group, the rumen contents of acetic acid and propionic acid were significantly increased in the EA group and reached the highest value at 2 h to 4 h after feeding (*p* < 0.05). Moreover, the relative abundances of specific rumen microbiota (Ruminococcaceae, *uncultured_rumen_bacterium, unclassified*_*Prevotella*, Bacteroidales, *Bacteroidota*, Bacteroidia, *unclassified_Rikenellaceae*, and *Prevotella_spBP1_145*) at the family and genus levels were significantly higher in the EA group (*p* < 0.05) compared to the CON group. The EA group exhibited significantly higher dry matter intake (*p* < 0.05) and increased the digestibility of neutral detergent fiber and ether extract when compared with the CON group (*p* < 0.05). Additionally, the plasma activities of total antioxidant capacity (T-AOC), superoxide dismutase (SOD), catalase (CAT), and glutathione peroxidase (GSH-Px) were significantly higher, while malondialdehyde (MDA) concentration was significantly lower in the EA group compared to the CON group (*p* < 0.05). In conclusion, dietary supplementation with 30 mg/kg BW EA in 5-month-old Kazakh sheep increased the dry matter intakQ16e, apparent digestibility of neutral detergent fiber, and ether extract, as well as the contents of acetic acid and propionic acid in rumen fluid. Moreover, EA supplementation regulated the ruminal microbiota, enhanced antioxidant capacity, and improved daily weight gain.

## 1 Introduction

In response to the antibiotic ban policy in animal husbandry, the quest for antibiotic alternatives in animal feed has gained momentum. Plant extracts stand out as vital options due to their medicinal attributes in enhancing animal health and productivity. Compared to conventional chemical drugs, plant extracts offer unique advantages in controlling inflammation and oxidative stress. In addition, they remarkably enhance animal health by bolstering immunity, satisfying healthy breeding standards to avert animal diseases, and improving animal production performance and product quality (1).

Ellagic acid (EA), a dimerized derivative of gallic acid widespread in various plants (2), has emerged as a potent alternative to antibiotics and possesses a variety of biological functions, such as antimutagenicity, antibacterial, anti-inflammatory, and antioxidant properties (3, 4). Xu et al. (5) evaluated the effect of gallic acid on calves, observing improved growth performance metrics, such as average daily gain, rumen fermentation parameters (total volatile fatty acids, propionate, and butyrate), and antioxidant levels (catalase and T-AOC). Notably, gallic acid reduced malondialdehyde and tumor necrosis factor-α levels in preweaning dairy calves while influencing ruminal microbial abundances of *Prevotellaceae_UCG-001*, Saccharofermentans, and *Prevotella_1*, while reducing the abundance of *Prevotella_7*. Gallic acid, a phenolic compound in plant extracts, demonstrates robust antioxidant capabilities, scavenging hydroxyl radicals, and exhibiting potent reducing power (6). Previous research has found that the addition of concentrated pomegranate peel extract (containing EA) to the diet of lactating dairy cows can prolong daily ruminating time and enhance the digestibility of dry matter, crude protein, neutral detergent fiber, cellulose, and hemicellulose (7). Moreover, concentrated pomegranate peel extract influences the relative abundance of methanogenic archaea, which are rumen-specific bacteria responsible for cellulose decomposition and lactic acid fermentation, and significantly improves the milk yield and growth performance of cattle (7). Studies involving monogastric animals have shown that dietary supplementation with EA can improve animal growth performance, intestinal health, and antioxidant capacity. Lu et al. found that feeding 500 g/t of EA to 30-day-old weaned piglets for 40 days increased the average daily gain and reduced diarrhea (8). Moreover, Qin et al. (9) demonstrated that the supplement of 0.1% EA to the basal diet of weaned piglets increased average daily feed intake and daily gain while reducing fecal score, thus suggesting an effect on intestinal bacteria. Additionally, it could alleviate oxidative stress and intestinal injury in weaned piglets (9). However, few studies have explored the effects of EA supplementation in ruminants.

EA primarily exists in the tannin form in nature (10). While traditionally considered antinutrients, low doses of plant-derived tannins enhance ruminant protein utilization (11). Condensed tannins bind to dietary crude protein, inhibiting its ruminal degradation. Consequently, this elevates rumen protein concentration, enhances amino acid utilization, and augments digestible protein throughout the digestive tract, ultimately improving production performance (12). Notably, limited studies have explored the effects of dietary supplementation of EA in sheep. In this study, 5-month-old Kazakh sheep received an EA-supplemented diet to investigate the effects of EA on growth performance, rumen metabolism, and apparent nutrient digestibility.

## 2 Materials and methods

### 2.1 Ethical considerations

All animal care and handling procedures in this study were conducted under the Guidance of the Care and Use of Laboratory Animals in China and were approved by the Animal Care Committee of Xinjiang Agricultural University of China (protocol permit number: 2020024).

### 2.2 Animal and experimental design

Ten 5-month-old Kazakh sheep (35.61 ± 2.32 kg, rams) of similar body weight (BW), well cared for, healthy, and equipped with rumen fistulas, were randomly assigned to two groups: the CON group (*n* = 5) and the EA group (*n* = 5) using a random number generator (http://www.r-project.org/). These sheep were housed in individual feeding pens (1.20 × 1.50 m) within a naturally ventilated barn structure. The ten pens were located inside a barn open on two sides and arranged in two rows of five, separated by the central feeding lane. The pens are enclosed by horizontal metal rail bars, which also delimit the pens at the feeding lane. The floor had a concrete base covered with barley straw bedding, of which one fresh flake (around 1.5 kg) per pen was added over the permanent bedding once a day. The sheep were untethered and did not have any access to a paddock area. The sheep in the CON group received a basal diet devoid of EA, whereas the diet for the EA group included EA supplementation at 30 mg/kg BW. The quality of the hay was checked according to guidelines in Cavallini et al. (13), ensuring the absence of molds and spores. The corn aflatoxin levels were assessed according to the procedure described in Girolami et al. (14) and were found to be below the maximum tolerable threshold recommended by the EU. Health checks, including fecal consistency and pH, as described below, were completed twice a week. The EA (≥90.00%) was purchased from Wufeng Chicheng Biotechnology Co., Ltd. (Hubei, China). The experimental sheep were raised at the 103 regiment experimental base of the 6^th^ division of the Xinjiang Production and Construction Corps in China. The feed was provided twice a day at 8:00 am and 8:00 pm, and the EA was weighed and mixed into the concentrate before feeding. The composition and ingredients of the basic diet are presented in Table 1. All sheep had *ad libitum* access to feed and clean water during the experiment. The experimental period lasted 30 days, preceded by a 5-day adaption stage.

**Table 1:** Composition and nutrient levels of the diet (DM basis).

**Items**	**Content**
Ingredients	
Yellow corn	35.00, %
Wheat bran	6.40, %
Soybean meal	10.00, %
Cottonseed meal	5.60, %
Premix^a^	3.00, %
Mixed hay (Alfalfa hay: Corn straw = 1:1)	40.00, %
Total	100.00
**Nutrient levels** ^b^	
Dry matter	87.56, %
Organic matter	95.25, %
Metabolic energy	11.26, MJ
Crude protein	16.78, %
Ether extract	2.86, %
Neutral detergent fiber	37.58, %
Acid detergent fiber	20.45, %
Calcium	1.02, %
Phosphorus	0.36, %

^a^ The premix provided the following per kg of the concentrate supplement: VA 10,000.00 IU, VD_3_ 2,550.00 IU, VE 20.00 IU, niacin 20.00 mg, biotin 0.06 mg, Cu (as copper sulfate) 22.00 mg, Fe (as ferrous sulfate) 94.00 mg, Mn (as manganese sulfate) 80.00 mg, Zn (as zinc sulfate) 88.00 mg, I (as potassium iodide) 0.75 mg, Se (as sodium selenite) 0.50 mg, Co (as cobalt chloride) 0.33 mg, Ca (as calcium carbonate and calcium hydrogen phosphate) 0.35%, P (calcium hydrogen phosphate) 0.125%, NaCl 0.80%; ^b^ Nutritional levels were measured.

### 2.3 Sample collection and analysis

The dry matter intake (DMI) of sheep was recorded daily. On the 21^st^ to 25^th^ day of the experiment, self-made fecal collection bags were used to collect fecal samples for 5 consecutive days. Specifically, the fecal samples were collected four times daily at fixed time intervals, meticulously documented, and pooled across each consecutive 5-day period. Subsequently, 10% of the total collected fecal amount was randomly selected, weighed, and dried for analysis. Feed and feces samples were sent to the Animal Nutrition Laboratory, College of Animal Science, Xinjiang Agricultural University, for dry matter (DM) and chemical analysis. To determine the DM content, the samples were dried in a forced air oven at 65°C until a constant weight was achieved. Upon drying, the samples were ground to pass through a 1 mm screen (Cyclotec Mill, model 1093; Foss Tecator, Höganäs, Sweden). The ash content of the ground samples was analyzed after 4 h of combustion in a muffle furnace at 550°C (Vulcan 3-550, Dentsply Ney-tech, Burlington, NJ, USA) ash-corrected α-amylase–treated neutral detergent fiber (NDF) with the addition of sodium sulfite (aNDFom) and acid detergent fiber (ADF) (15); Crude protein (CP) using a Kjeldahl nitrogen analyzer (Gerhardt Vapodest 50, Gerhardt GmbH, Königswinter, Germany); Starch (16), method 996.11; and Ether extract (EE; according to EC Regulation No. 152/2009). Further, the calcium (Ca) and phosphorus (P) contents of feed and feces were analyzed by atomic absorption spectrophotometer (17). Average daily gain (ADG) was calculated as the difference between final and initial body weight divided by the number of days of feeding. Feed/Gain (F/G) was calculated as the ratio of average daily feed intake to daily weight change (g/g).

Rumen fluid samples were collected on the 15^th^ and 30^th^ day of the trial period, both before feeding (0 h) and at 2, 4, 6, and 8 h after feeding from the same position of the fistula with a self-made rumen fluid collector. Fluid sampling devices consisted of Tygon tubing terminating in a pot scrubber, weighted with several steel nuts installed in each animal, and were threaded through holes in the cannula plug to maintain the anaerobic rumen environment. One pot scrubber was placed in the cranial portion of the rumen, and one was placed in the caudal portion. The ends of the Tygon tubing were scored to allow a lure lock syringe to be screwed directly onto each tube. For each rumen fluid sample collected, a 50 mL syringe was then used to sample equal volumes of fluid from each sampling line. Samples were mixed in the syringe, and the bulk sample was aliquoted into 2 glass vials (18), filtered with a 60-mesh nylon bag, and packed into frozen tubes immediately after pH was measured by a portable pH meter (PB-21, Sartorius, Germany), and preserved in liquid nitrogen for the subsequent rumen fermentation parameter analysis including the volatile fatty acids (VFAs) and ammonia nitrogen (NH_3_-N) (Agilent Cary 60UV-Vis Spectrophotometer, USA) by gas chromatography (colorimetry).

Blood samples were collected from the jugular vein on the 30^th^ day of the experiment at 07:30 am into tubes containing an anticoagulant (heparin lithium). The samples were centrifuged at 3,500 × *g* for 15 min to collect plasma and stored at −20°C. The Catalase (CAT; #A005), superoxide dismutase (SOD; #A001-1), glutathione peroxidase (GSH-PX, #A007-2), total antioxidant capacity (T-AOC, #A015), and malondialdehyde (MDA, #A003-1) were determined with the commercial test kit procured from Nanjing Jiancheng Biotechnology Research Institute (Nanjing, China).

### 2.4 16S rDNA sequencing and bioinformatics analysis of the rumen bacteria

Total DNA extraction and PCR amplification of rumen fluid samples followed the methodology outlined in Ma et al.'s study (19). Briefly, total DNA extraction involved the use of cetyltrimethylammonium bromide (CTAB), with subsequent assessment of DNA purity and concentration via 1% agarose gel electrophoresis and spectrophotometry. Universal prokaryotic primers 341F (5′-CCTACGGGNGGCWGCAG-3′) and 806R (5′-GGACTACHVGGGTATCTAAT-3′) were used to amplify the V3-V4 variable region of bacterial 16S rRNA gene. The amplifiers were sequenced on the Miseq PE300 platform (Illumina, USA). The 16S rRNA gene sequencing raw reads were qualitatively filtered using Flash (version 1.20) and QIIME (Quantitative Insight into Microbial Ecology, version 1.8.0) (20). The filtered sequences were compared using homologous clusters to obtain the operational taxonomic units (OTUs). The α and β diversity indices were measured and analyzed. Linear discriminant analysis (LEfSe) of effect size was used to identify differential microflora, and PICRUSt analysis was performed to predict microbial function.

### 2.5 Statistical analysis

Preliminary analysis of the experimental data was conducted using Excel 2010. The normality (Shapiro-Wilk) test was performed prior to the statistical analysis. The data of growth performance, rumen fermentation, nutrient apparent digestibility, and plasma antioxidant were analyzed for normality using the Shapiro–Wilk test, and further statistical analysis was carried out using SPSS 20.0 (SPSS Statistics 20, IBM Japan, Ltd., Tokyo, Japan) software using independent sample *t-*tests. The data were expressed as mean ± standard deviation, with *p* < 0.05 indicating significant differences and 0.05 < *p* < 0.10 indicating a significant trend of differences. Pearson's correlation analysis was performed to evaluate the correlation between rumen differential bacteria and rumen fermentation parameters and apparent digestibility of nutrients, and graphs were rendered using Orgin 8.0 (OriginLab Co., Northampton, MA, USA).

## 3 Results

### 3.1 Growth performance

Table 2 demonstrates that the EA group sheep exhibited increased final body weight, average daily gain, and feed conversion efficiency compared to sheep in the CON group. However, no statistically significant differences were observed between the CON and EA groups (*p* > 0.05).

**Table 2:** Effect of the dietary addition of ellagic acid on growth performance of Kazakh sheep.

**Item**	**CON group**	**EA group**	**p-value**
Initial body weight, kg	35.33 ± 2.83	35.88 ± 2.29	0.744
Final body weight, kg	41.61 ± 2.33	43.14 ± 2.52	0.347
Average daily gain, g/sheep·d	209.33 ± 21.43	242.00 ± 31.98	0.094
Feed:Gain, g/g	4.82 ± 0.55	4.30 ± 0.51	0.159

CON group, fed a basal diet; EA group, fed a basal diet with EA (30 mg/kg BW).

### 3.2 Rumen fermentation parameters

Rumen fermentation parameters on Day 15 and Day 30 are presented in Table 3. No significant differences were observed in rumen pH value and isobutyric acid, butyric acid, isovaleric acid, valeric acid, and ammonia nitrogen contents between the EA and CON groups (*p* > 0.05). Nevertheless, the contents of acetic acid (*p* = 0.003) and propionic acid (*p* = 0.003) exhibited significant increases, while lactic acid (*p* = 0.086) contents tended to be higher in the EA group compared to the CON group. Specifically, on the 30^th^ day, acetic acid content in the EA group increased by 10.32% compared to the CON group (*p* = 0.056), although other parameters showed no significant differences.

**Table 3:** Effect of the dietary addition of ellagic acid on rumen fermentation parameters of Kazakh sheep.

**Item**	**CON group**	**EA Group**	**p-value**
Day 15			
pH	6.67 ± 1.16	6.68 ± 0.30	0.946
Acetic acid, mmol/L	74.88 ± 0.88^b^	77.56 ± 1.12^a^	0.003
Propionic acid, mmol/L	18.08 ± 0.51^b^	21.37 ± 1.71^a^	0.003
Isobutyric acid, mmol/L	0.38 ± 0.05	0.37 ± 0.04	0.567
Butyric acid, mmol/L	13.20 ± 0.89	13.10 ± 0.91	0.863
Isovaleric acid, mmol/L	0.63 ± 0.06	0.58 ± 0.07	0.314
Valeric acid, mmol/L	0.75 ± 0.05	0.74 ± 0.05	0.966
Ammonia nitrogen, mmol/L	14.87 ± 2.38	14.28 ± 2.16	0.689
Lactic acid, mmol/L	2.92 ± 0.27	3.43 ± 0.43	0.086
Day 30			
pH	6.33 ± 0.17	6.52 ± 0.36	0.320
Acetic acid, mmol/L	79.24 ± 6.13	87.42 ± 5.42	0.056
Propionic acid, mmol/L	24.37 ± 4.04	26.70 ± 4.12	0.337
Isobutyric acid, mmol/L	0.54 ± 0.02	0.57 ± 0.05	0.249
Butyric acid, mmol/L	15.19 ± 1.95	17.87 ± 3.15	0.145
Isovaleric acid, mmol/L	1.01 ± 0.05	1.03 ± 0.25	0.836
Valeric acid, mmol/L	1.02 ± 0.19	1.34 ± 0.40	0.147
Ammonia nitrogen, mmol/L	17.49 ± 2.08	19.99 ± 4.10	0.258
Lactic acid, mmol/L	4.94 ± 0.29	5.20 ± 0.22	0.151

^a, b^ Values within a row without common superscripts differ significantly (*p* < 0.05); CON group, fed a basal diet; EA group, fed a basal diet with EA (30 mg/kg BW).

**Figure 1:**
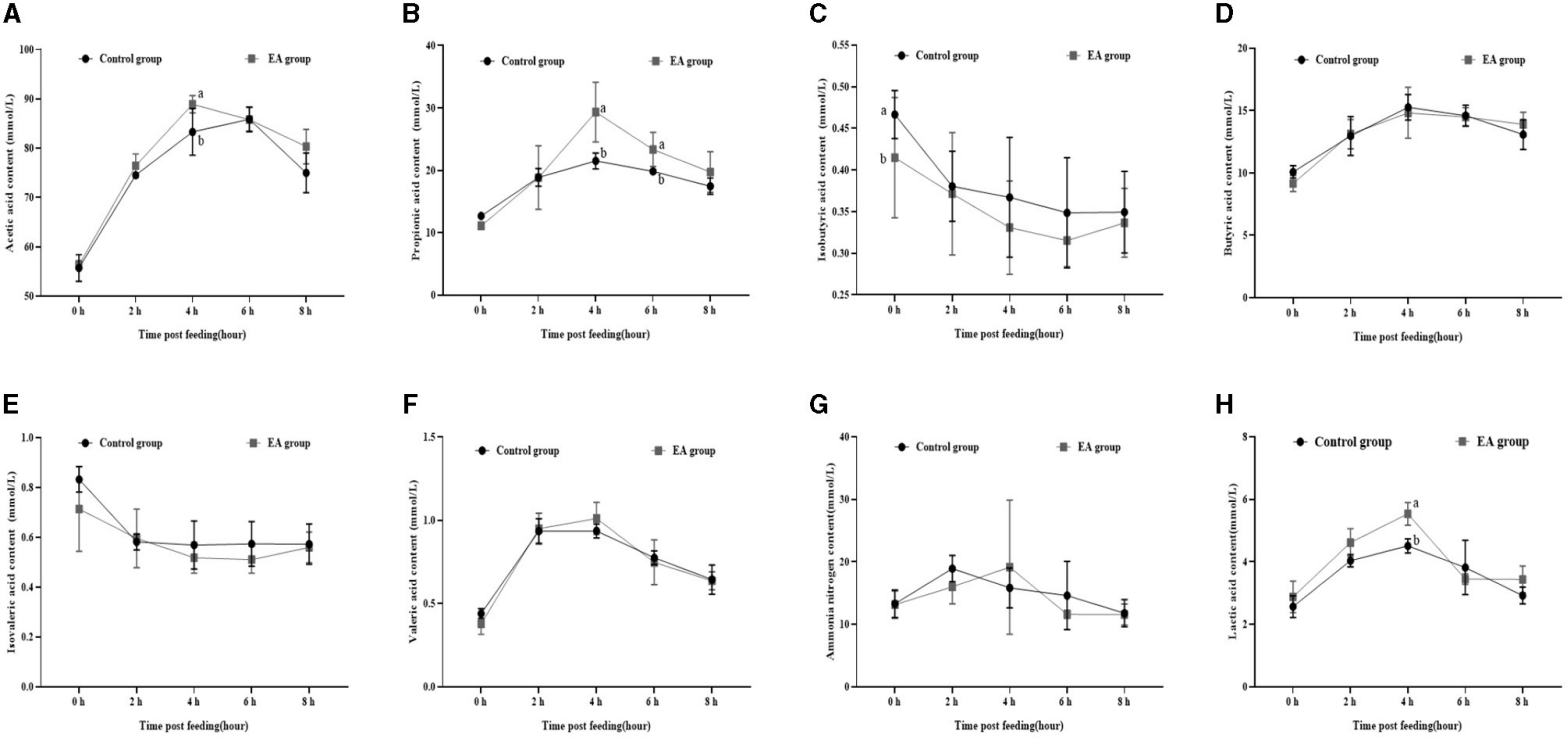
Effect of the dietary addition of ellagic acid on the rumen fermentation dynamics of Kazakh sheep (on day 15). ^a, b^ Values within a row without common superscripts differ significantly (*p* < 0.05); CON group, fed a basal diet; EA group, fed a basal diet with EA (30 mg/kg BW). **(A)** Acetic acid, **(B)** Propionic acid, **(C)** Isobutyric acid, **(D)** Butyric acid, **(E)** Isovaleric acid, **(F)** Valeric acid, **(G)** Ammonia nitrogen, and **(H)** Lactic acid.

As shown in Figure 1, on the 15^th^ day of the experiment, at 1 h after morning feeding, the rumen fluid contents of acetic acid (Figure 1A), propionic acid (Figure 1B), and lactic acid (Figure 1H) in the EA group reached the maximum value, which was higher (*p* < 0.05) than that of the CON group, and then began to decrease. The contents of butyric acid (Figure 1D) and valeric acid (Figure 1F) showed the same trend. However, no significant difference was observed between the EA and CON groups (*p* > 0.05). The contents of isobutyric acid (Figure 1C) and isovaleric acid (Figure 1E) in the rumen fluid of the two groups began to decrease after feeding.

**Figure 2:**
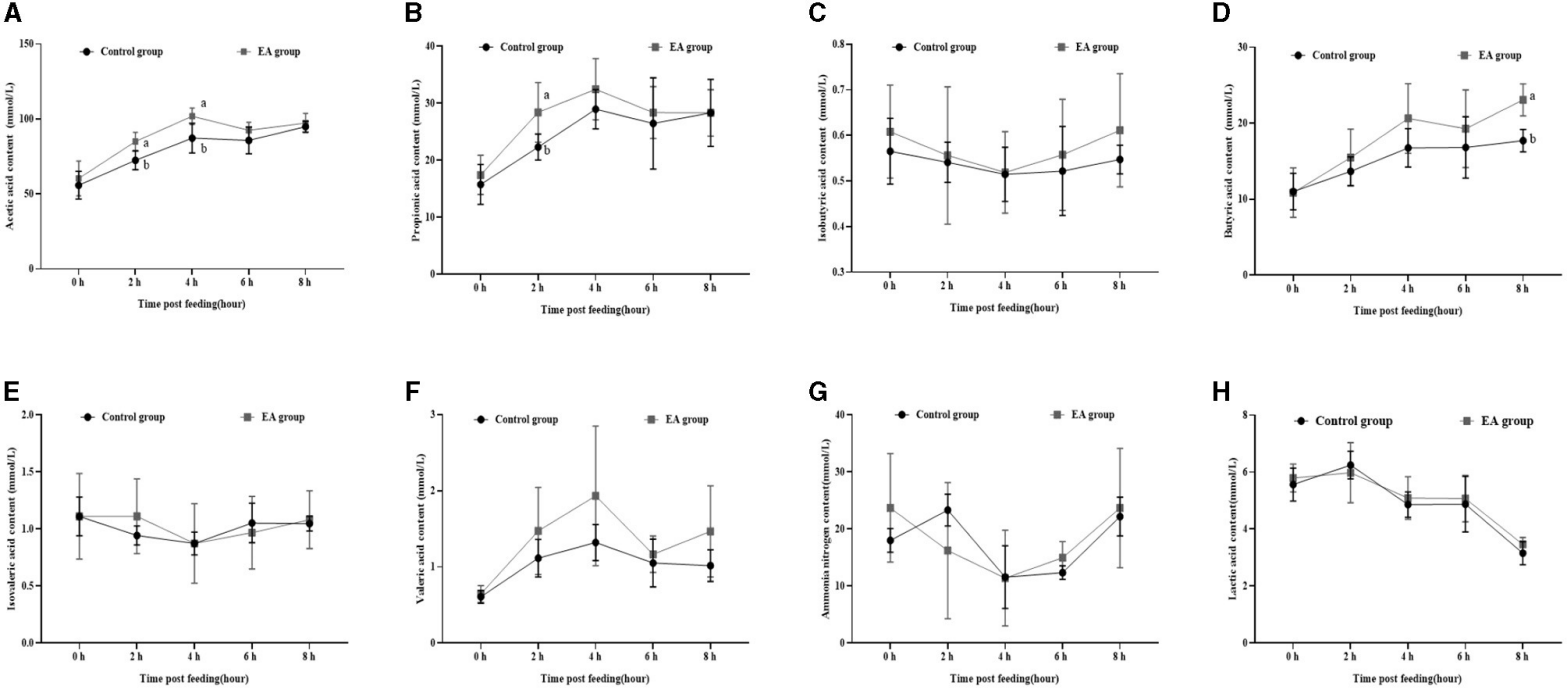
Effect of the dietary addition of ellagic acid on the rumen fermentation dynamics of Kazakh sheep (on day 30). ^a, b^ Values within a row without common superscripts differ significantly (*p* < 0.05); CON group, fed a basal diet; EA group, fed a basal diet with EA (30 mg/kg BW). **(A)** Acetic acid, **(B)** Propionic acid, **(C)** Isobutyric acid, **(D)** Butyric acid, **(E)** Isovaleric acid, **(F)** Valeric acid, **(G)** Ammonia nitrogen, and **(H)** Lactic acid.

As shown in Figure 2, on the 30^th^ day of the experiment, the changing trend of acetic acid (Figure 2A), propionic acid (Figure 2B), isobutyric acid (Figure 2C), butyric acid (Figure 2D), isovaleric acid (Figure 2E), valeric acid (Figure 2F), and ammonia nitrogen (Figure 2G) in rumen fluid of the two groups was similar to that on the 15^th^ day, whereas lactic acid content showed the opposite trend (Figure 2H). Moreover, 2 h after morning feeding, the contents of acetic acid and propionic acid in the rumen fluid of the EA group were significantly higher than those of the CON group (*p* < 0.05).

### 3.3 Rumen bacterial diversity

**Figure 3:**
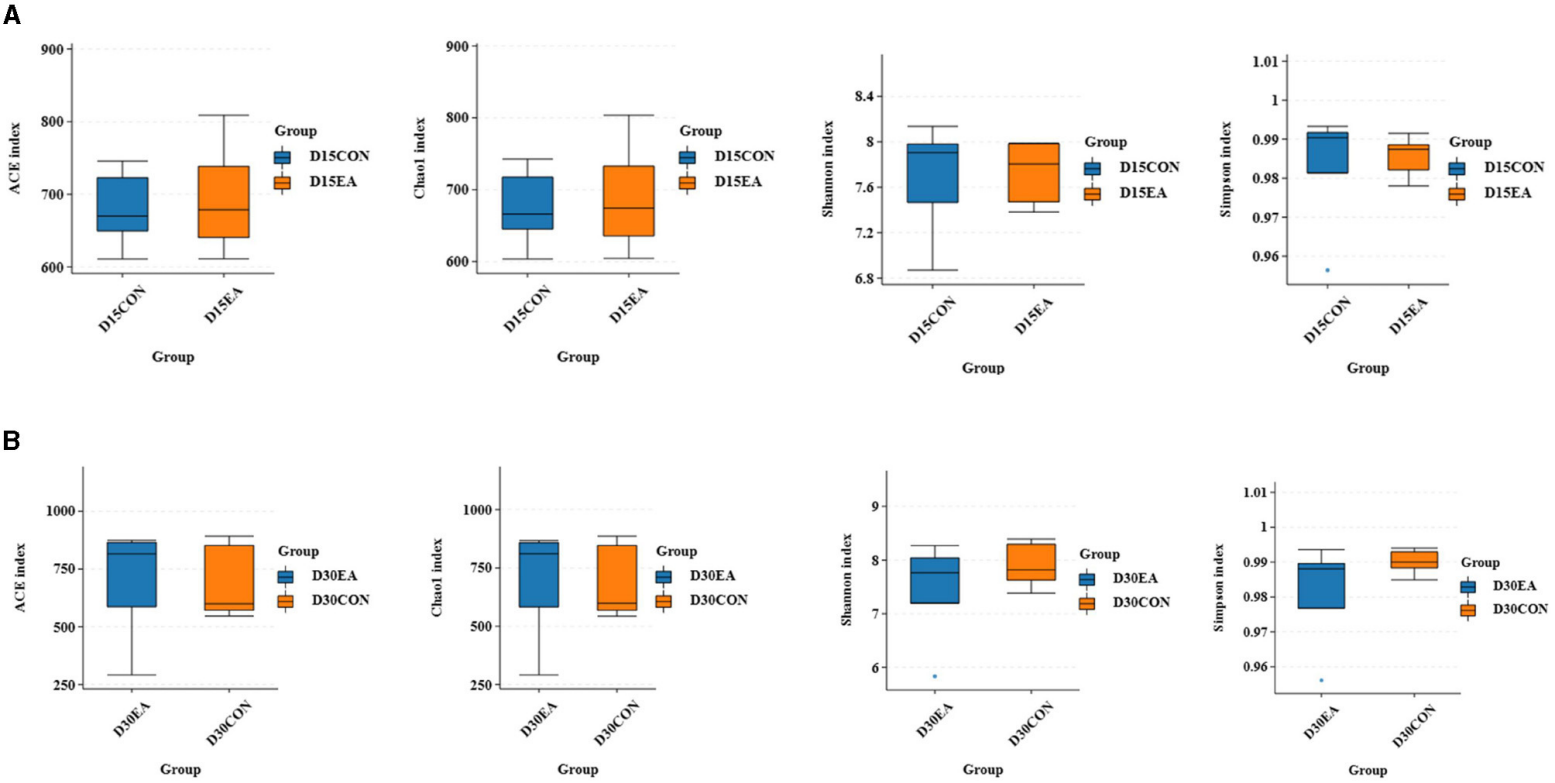
The results of α diversity analysis. **(A)** The ACE, Chao1, Shannon, and Simpson indices of rumen microorganisms on the 15^th^ day of the experiment. **(B)** The ACE, Chao1, Shannon, and Simpson indexes of rumen microorganisms on the 30^th^ day of the experiment.

**Figure 4:**
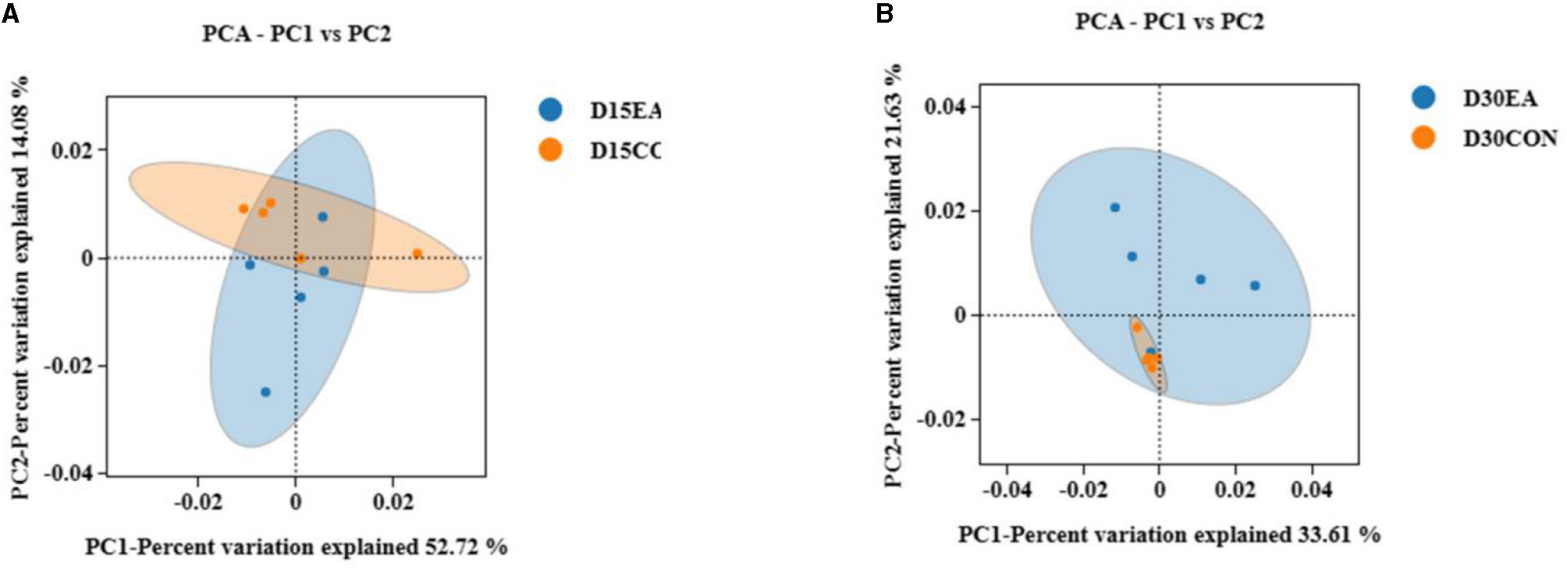
The results of β diversity analysis. **(A)** Weighted Unifrac PCoA scatter plot on the 15^th^ day of the experiment. **(B)** Weighted Unifrac PCoA scatter plot on the 30^th^ day of the experiment.

An average of 65,767 effective labels per sample were obtained on the 15^th^ day. By the 30^th^ day, an average of 678 OTUs per sample were acquired with 97% paired sequence identity. Additionally, an average of 66,514 effective tags per sample was obtained, resulting in an average of 692 OTUs per sample with 97% paired sequence identity. ACE, Chao1, Shannon, and Simpson indices exhibited no significant differences between the CON and EA groups (Figures 3A, B). The intestinal microbiomes of both groups exhibited wide distribution and effective isolation, suggesting EA's impact on rumen microflora composition (Figure 4).

**Figure 5:**
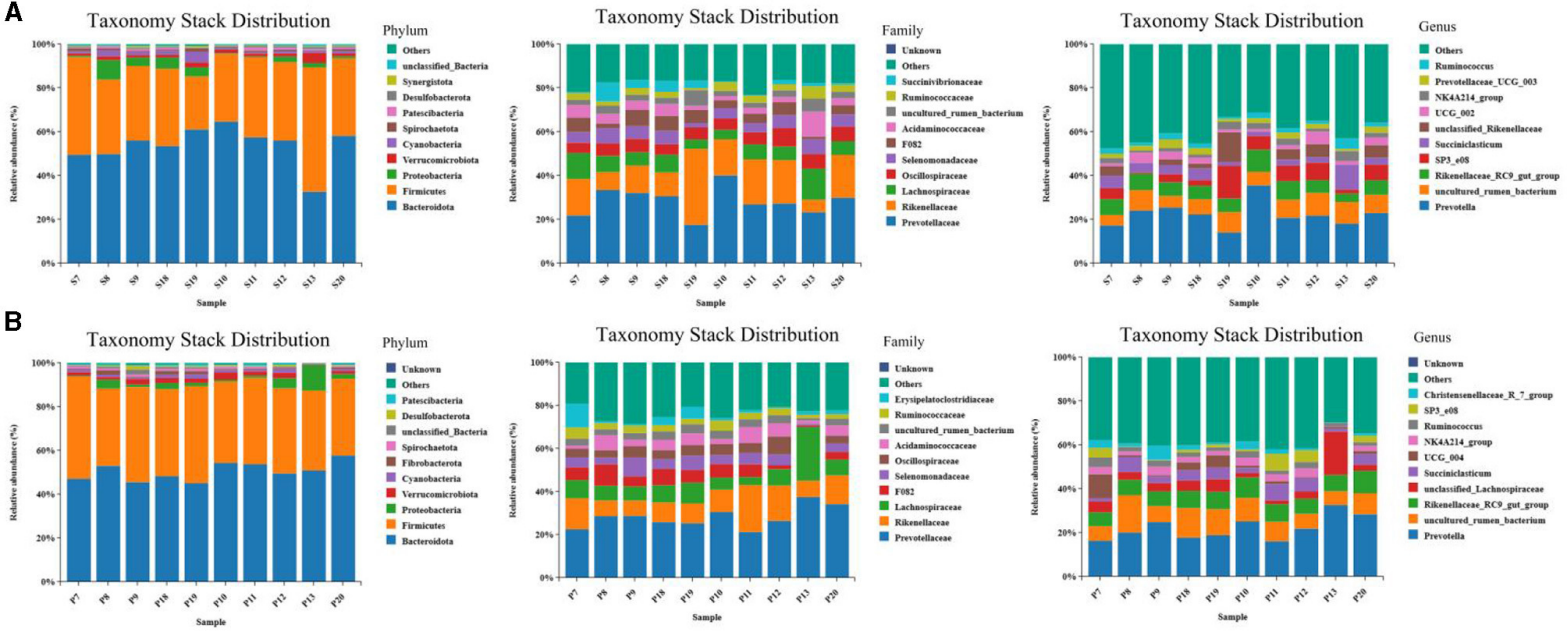
Taxonomic and stack distribution of different species. **(A)** Taxonomic stack distribution at phylum, family, and genus levels on the 15^th^ day of the experiment. **(B)** Taxonomic stack distribution at gate, family, and genus levels on the 30^th^ day of the experiment.

The results of the microbiome composition analysis are presented in Figure 5. On the 15^th^ day, Bacteroidetes and Firmicutes were the dominant phyla in the rumen of the two groups, accounting for over 85% (*p* < 0.05) of microflora. At the family level, Prevotellaceae and Rikenellaceae were the dominant families in the rumen of the two groups, while at the genus level, the abundance of *Prevotella, uncultured_rumen_bacterium*, and other bacteria in the rumen of sheep in the EA group was marginally but insignificantly higher than that in the CON group (*p* > 0.05) (Figure 5A). On the 30^th^ day, at the phylum level, Bacteroides and Streptomyces were the most predominant phyla of the two groups of the rumen, accounting for more than 90% of all microorganisms; at the family level, the abundance of Erysipelatoclostridiaceae in the rumen of the EA group was significantly higher than that of the CON group (*p* < 0.05); at the genus level, the abundance of *SP3_e08* in the rumen of EA group was higher than that of CON group, while the abundance of *UCG_004* was significantly lower than that in the CON group (*p* < 0.05) (Figure 5B).

**Figure 6:**
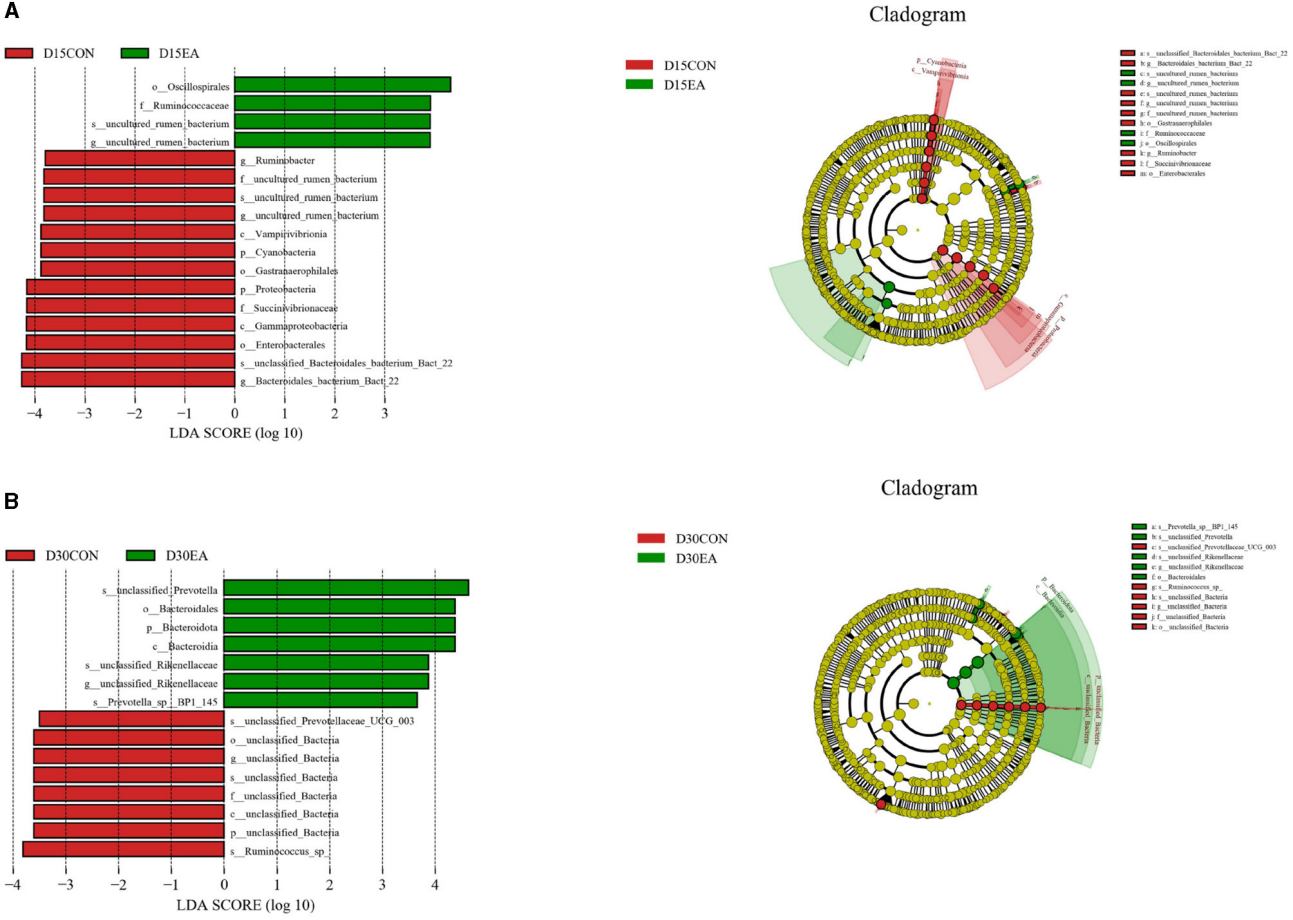
LEfSe analysis of rumen microflora. **(A)** LEfSe analysis results on the 15^th^ day of the experiment. **(B)** LEfSe analysis results on the 30^th^ day of the experiment. D15CON, D30CON, fed a basal diet; E15 EA, E30 EA, fed a basal diet with EA (30 mg/kg BW).

LEfSe analysis was used to compare the microbiota in the rumen contents of the two groups. On the 15^th^ day of the experiment, the abundances of Oscillospirales, Ruminococcaceae, uncultured_rumen_bacterium, and *uncultured rumen bacterium* in the rumen of the EA group were higher than those in the CON group (*p* < 0.05) (Figure 6A). On the 30^th^ day of the experiment, the rumen abundances of unclassified_Prevotella, Bacteroidales, *Bacteroidota*, Bacteroidia, *Unclassified_Rikenellaceae, unclassified_Rikenellaceae*, and *Prevotella_spBP1_145* in the EA group were higher than those in the CON group (*p* < 0.05) (Figure 6B).

**Figure 7:**
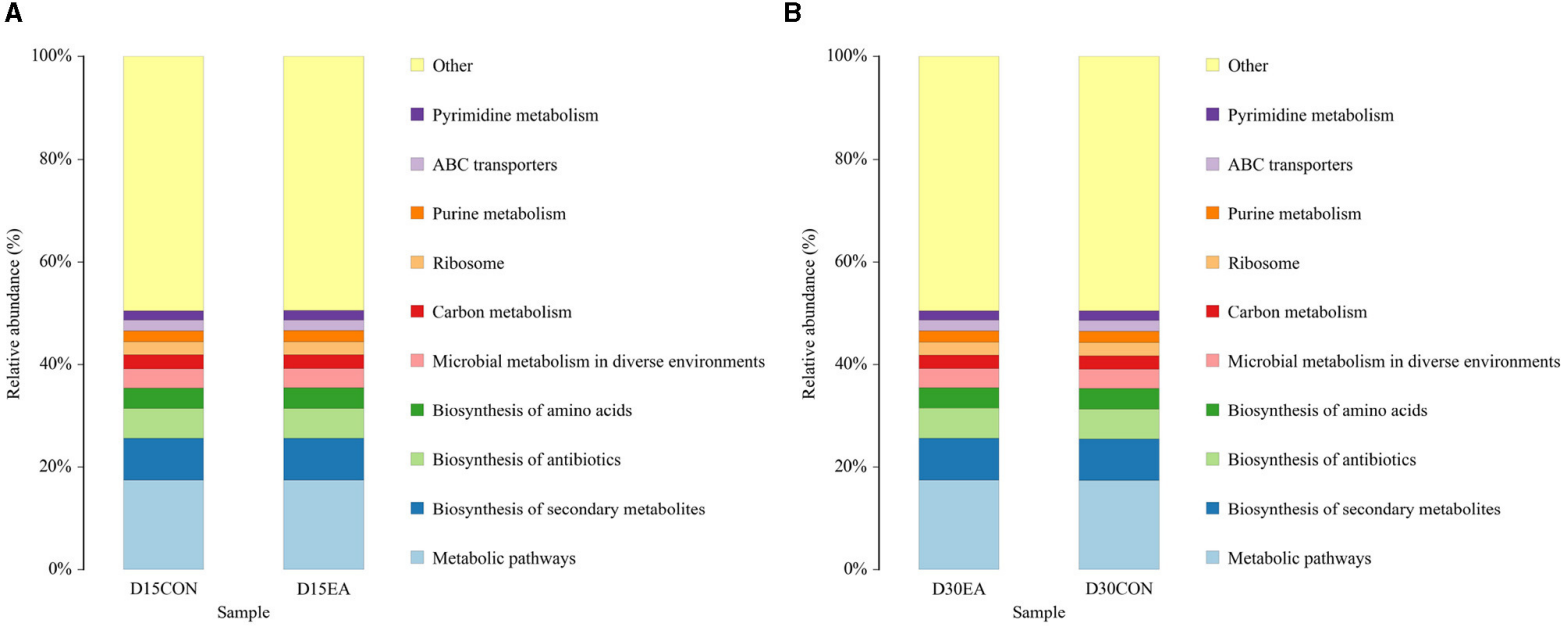
PICRUSt function prediction chart. **(A)** The 15^th^ day of the experiment. **(B)** The 30^th^ day of the experiment. D15CON, D30CON, fed a basal diet; E15 EA, E30 EA, fed a basal diet with EA (30 mg/kg BW).

PICRUSt analysis revealed comparable functions of rumen microbiota in both groups, primarily associated with metabolic pathways, biosynthesis of secondary metabolites, antibiotics, amino acids, and microbial metabolism (Figure 7).

### 3.4 Apparent nutrient digestibility

As shown in Table 4, compared with the CON group, the intake of DM and OM of sheep in the EA group increased significantly (*p* < 0.05). Additionally, the apparent digestibility of NDF and EE were increased significantly by 6.12% and 3.17% (*p* < 0.05).

**Table 4:** Effect of the dietary addition of ellagic acid on apparent nutrient digestibility of Kazakh sheep.

**Item**	**CON group**	**EA Group**	**p-value**
Intake, g/day			
Dry matter	1,253.68 ± 52.31^b^	1,321.81.67 ± 30.37^a^	0.036
Organic matter	1,194.18 ± 49.83^b^	1,259.07 ± 28.93^a^	0.036
Apparent digestibility, %			
Dry matter	67.58 ± 1.31	68.11 ± 1.00	0.487
Organic matter	68.91 ± 1.38	69.99 ± 0.94	0.188
Crude protein	64.98 ± 3.88	66.99 ± 1.01	0.294
Neutral detergent fiber	60.02 ± 1.96^b^	63.44 ± 1.34^a^	0.020
Acid detergent fiber	60.97 ± 1.35	61.84 ± 4.95	0.713
Ether extract	68.71 ± 2.01^b^	70.89 ± 1.38^a^	0.044
Calcium	47.80 ± 0.53	48.50 ± 2.05	0.483
Phosphorus	44.63 ± 5.70	45.04 ± 3.01	0.888

^a, b^ Values within a row without common superscripts differ significantly (p < 0.05); CON group, fed a basal diet; EA group, fed a basal diet with EA (30 mg/kg BW).

### 3.5 Plasma antioxidant capacity

As shown in Table 5, the activities of T-AOC, SOD, CAT, and GSH-Px in the plasma of the EA group were significantly higher than those in the CON group (*p* < 0.05), and the content of MDA decreased significantly (*p* < 0.05).

**Table 5:** Effect of the dietary addition of ellagic acid on plasma antioxidant capacity of Kazakh sheep.

**Item**	**CON group**	**EA Group**	**P-value**
T-AOC, U/mL	0.69 ± 0.01^b^	0.74 ± 0.02^a^	0.007
SOD, U/mL	123.48 ± 4.99^b^	134.79 ± 2.70^a^	0.002
CAT, U/mL	0.54 ± 0.11^b^	0.88 ± 0.10^a^	0.001
GSH-Px, U/mL	75.95 ± 5.61^b^	87.95 ± 7.41^a^	0.020
MDA, nmol/mL	3.36 ± 0.20^a^	3.01 ± 0.17^b^	0.018

^a, b^ Values within a row without common superscripts differ significantly (*p* < 0.05); CON group, fed a basal diet; EA group, fed a basal diet with EA (30 mg/kg BW).

### 3.6 Correlation analysis between rumen differential bacteria and rumen fermentation parameters, apparent digestibility of nutrients, and plasma antioxidant capacity

**Figure 8:**
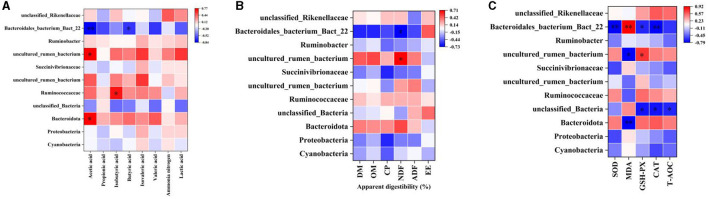
Correlation analysis. **(A)** Rumen differential bacteria and Rumen fermentation parameters. **(B)** Rumen differential bacteria and apparent digestibility of nutrients. **(C)** Rumen differential bacteria and plasma Antioxidant capacity. “*” indicates a significant difference between groups where *p* < 0.05. “**” indicates a significant difference between groups where *p* < 0.01.

The correlation between rumen differential bacteria and rumen fermentation parameters, apparent digestibility of nutrients, and plasma antioxidant capacity was explored. Acetic acid content in the rumen was positively correlated with uncultured_rumen_bacterium and *Bacteroidota* abundance, and significantly negatively correlated with *Bacteroidales_bacterium_Bact_22*. Isobutyric acid content was positively correlated with Ruminococcaceae abundance, and Butyric acid content was negatively correlated with *Bacteroidales_bacterium_Bact_22* (Figure 8A). The apparent digestibility of NDF was positively correlated with rumen uncultured_rumen_bacterium abundance and negatively correlated with *Bacteroidales_bacterium_Bact_22* (Figure 8B). *Bacteroidales_bacterium_Bact_22* abundance was negatively correlated with SOD, GSH-Px, and CAT activity and positively correlated with MDA content; uncultured_rumen_bacterium abundance was negatively correlated with MDA content and positively correlated with GSH-Px; unclassified_Bacteria abundance was negatively correlated with GSH-Px, CAT activity, and T-AOC; Bacteroidota was negatively correlated with MDA content (Figure 8C).

## 4 Discussion

EA exerts various biological functions, including anti-oxidative, anti-cancer, and anti-inflammatory properties, which have spurred substantial research interest in its practical applications (21). At present, there are few reports on the effect of EA on ruminants. To better understand the effects of dietary EA supplementation in sheep, in the present experiment, we used 10 ruminally cannulated Kazakh sheep to assess the effects of dietary addition of EA on growth performance, rumen metabolism, and apparent nutrient digestibility. The DMI required for ruminants to maintain their life activities determines the quality of their growth, development, and reproduction (22). In this study, we found that dietary supplementation with EA had a tendency to increase ADG (*p* = 0.094), indicating its beneficial effect on growth performance in sheep. The underlying cause may be the higher DMI intake of sheep in the EA group. This suggests that the inclusion of EA in the diet could have positive effects on palatability, and consequently, on feed intake. Orzuna-Orzuna et al. evaluated the effects of dietary tannin supplementation on performance, carcass characteristics, meat quality, oxidative stability, and serum antioxidant capacity of sheep by meta-analysis. The results showed that dietary tannin supplementation did not affect the dry matter intake of sheep but increased the daily gain (23). These findings are similar to those of this study. It could be presumed that tannins reduce the consumption of microbial protein, improve the efficiency of microbial protein synthesis, promote protein flow to the duodenum, and thus improve the production performance of ruminants by inhibiting ciliated protozoa in the rumen (24). Unfortunately, rumen protozoa were not studied in the current research. We plan to investigate this in future studies.

Many plants rich in secondary metabolites or bioactive compounds can affect the growth or activity of rumen microorganisms through different mechanisms to regulate rumen fermentation characteristics (25). The change in pH of rumen fluid is a comprehensive reflection and intuitive manifestation of the change in the internal environment of rumen fermentation. Extreme pH values adversely affect the growth and reproduction of rumen microorganisms and feed substrate fermentation (26). In this study, the rumen fluid pH values of the two groups were in the normal range (6.33–6.68) without significant differences, which is consistent with the previous studies (27). Rumen NH_3_-N concentration is not only one of the main internal environmental indicators of rumen fermentation but also the most important N source of microbial protein synthesis in rumen (28). The increase of rumen VFAs and NH_3_-N production generally indicates the improvement of rumen microbial metabolic activity, nitrogen use efficiency, and overall productivity of ruminants (29, 30). In this study, after the dietary supplementation of EA, we found no significant increase in rumen ammonia nitrogen concentration, except for improved rumen VFAs (acetic acid and propionic acid significantly increased). Manoni et al. used a short-term *in vitro* rumen fermentation model to better understand the effects of EA and gallic acid on rumen fermentation and discovered that EA had a significant effect on reducing CH_4_ emission and ammonia formation as well as affecting rumen degradability and total SCFA yield (31), which is consistent with the results of our study. Regrettably, we did not measure the CH_4_ of the rumen, limiting the scope of inquiry. Xu et al. (5) evaluated the effect of Gallic acid on rumen fermentation of pre-weaning calves. The results showed that the concentrations of total volatile fatty acids, propionate, butyrate, and valerate in the rumen fluid of calves increased linearly with the addition of gallic acid, resulting in a linear decrease in pH (5). While EA might have a dose-dependent effect in this experiment, since only a single EA concentration was used in this study, follow-up research is required to investigate this aspect. Bhatta et al. studied the effect of tannin on rumen fermentation *in vitro* and found that different addition of tannin could reduce the average NH_3_-N concentration (32). However, the addition of tannin to the diet of dairy cows did not affect the concentration of NH_3_-N (33). The differences in the above results may be related to the source, type, and molecular weight of tannins.

Furthermore, our assessment of rumen fermentation dynamics in Kazakh sheep revealed a pattern: initially, the levels of acetic acid, propionic acid, butyric acid, pentanoic acid, and lactic acid increased before gradually declining, reaching their peak at the 4-hour mark post-feeding (refer to Figures 1, 2).

Large and diverse rumen microflora play a key role in the growth and health of ruminants. There is increasing evidence that concentrated tannins from various plants or plant extracts have a significant effect on the rumen microflora of ruminants and can selectively change specific rumen bacteria, thus altering the metabolism of volatile fatty acids (VFAs) in the rumen (34). The effect of EA on rumen microorganisms has not been reported. Herein, we performed 16S rDNA high-throughput sequencing to detect the effect of dietary EA on the composition of rumen microflora of sheep and found that the trend of microbial diversity was consistent between the two groups of sheep rumen samples (Figure 3). Secondly, whether in the CON group or EA group, Bacteroides and Streptomyces were the dominant phyla in the rumen of Kazakh sheep, which was consistent with the findings of other ruminants (35, 36), and there was no significant change in the composition of the top 10 dominant families and genera between the two groups (Figure 5). However, dietary EA could regulate the abundance of rumen microorganisms. For example, after the addition of EA to the diet, the abundance of Cyanobacteria and Synergistota in the rumen of sheep in the EA group decreased. Synergistota has been found in a wide range of anaerobic environments, and some members are associated with amino acid transport (37). Cyanobacteria is a common rumen bacterial phylum, which plays a vital role in hemicellulose and pectin degradation and methane production reduction (38). The change in the relative abundance of Cyanobacteria in the rumen may be driven by the change in feed quality and their ability to degrade plant hemicellulose and pectin. The deficiency of our study is also the lack of determination of amino acid content and methane production. Therefore, the effects of dietary EA on energy utilization and amino acid fermentation in sheep need to be further studied. LEfSe was used to classify the characteristics of microflora with rich differences among animal subgroups. In this experiment, two groups of sheep rumen differential bacteria were compared. Results showed that the abundances of Ruminococcaceae, *uncultured_rumen_bacterium, Prevotella*, and *SP3_e08* in the EA group were significantly higher than those in the CON group (Figure 6). The increase in Ruminococcaceae abundance could be attributed to the inclusion of EA in the diet, potentially enhancing the cellulose and hemicellulose degradation capabilities in Kazakh sheep. This family possesses a significant quantity of hemicellulase and oligosaccharide degradase enzymes, which might explain this observed change (39). Subsequently, we analyzed the correlation between rumen differential bacteria and rumen fermentation parameters and found that dietary supplementation with EA could significantly increase the rumen Ruminococcaceae abundance in sheep. Moreover, we observed a significant positive correlation between the bacteria and isobutyric acid content. Therefore, the increase of rumen cocci abundance in sheep may be responsible for the increase in TVFA production in our study. In addition, we found that the significantly upregulated bacterial *Bacteroidales_bacterium_Bact_22* in the rumen of sheep in the CON group had a significant negative correlation with acetic acid and butyric acid content. In the EA group, the significantly upregulated bacterial *uncultured_rumen_bacterium* was positively correlated with acetic acid content, and *Bacteroidota* content was positively correlated with acetic acid content. Our findings suggest that dietary supplementation of ellagic acid can improve the rumen fermentation of Kazakh sheep by regulating the abundance of rumen microorganisms, which has a beneficial effect on growth performance.

Dietary nutrient digestibility is another important parameter for evaluating dietary utilization rate (40). The improvement of animal growth performance is related to the high digestibility of diet. After adding EA to the diet, we observed that the digestibility of NDF increased significantly, and the digestibility of dry matter and crude protein showed a noticeable but insignificant increase (Table 3). In a previous study, digestibility was improved with the addition of 30 mg/kg BW/d EA to equine diets for various components including DM, OM, gross energy, NDF, ADF, and Ca (41). Hence, based on the results of the current study, sheep supplemented with EA had a high potential to improve the digestibility of DM and nutrients. Additionally, we analyzed the correlation between rumen differential bacteria and apparent nutrient digestibility. The results showed that the apparent digestibility of NDF was positively correlated with the upregulated bacterial *uncultured_rumen_bacterium* in the rumen of sheep in the EA group and negatively correlated with the digestibility of NDF in the rumen of sheep in the CON group. In terms of apparent digestibility, the apparent digestibility of NDF in the EA group was significantly higher than that in the CON group (Figure 8B). It can be concluded that the dietary supplementation of EA can improve the apparent digestibility of Kazakh sheep, which may be related to the upregulation of rumen *uncultured_rumen_bacterium* bacteria.

EA can inhibit oxidative stress by directly scavenging free radicals, inhibiting lipid peroxidation, increasing the activity of antioxidant enzymes and gene expression, maintaining cell stability, and reducing DNA damage by regulating SOD, MDA, CAT, and GSH-Px levels in the blood (42). We evaluated the effect of dietary EA on the plasma antioxidant indices of Kazakh sheep. The results showed that dietary EA could significantly increase the activities and T-AOC of SOD, CAT, GSH-Px, and other enzymes, and decrease the MDA content (Table 4). Previous studies in piglets (9), mice (43), and broilers (44) have highlighted the antioxidant effect of EA, which is consistent with the results of our experimental study. Moreover, changes in the microbiota are linked to alterations in the redox state (45). A previous study (46) found that antioxidants can regulate the dynamic balance of intestinal microbiota by scavenging excessive free radicals and strengthening organic immunity. Furthermore, some studies have indicated that *Lactobacillus* and *Bifidobacterium* possess excellent antioxidant capacity by scavenging free radicals (47, 48). In our experiment, the dietary supplementation of ellagic acid improved the antioxidant capacity of sheep and regulated the ruminal microbiota. Hence, we analyzed the correlation between rumen differential bacteria at the phylum, family, and genus level, and plasma antioxidant capacity. The analysis unveiled significant associations, such as the positive correlation of *uncultured_rumen_bacterium* abundance in the EA group with GSH-Px activity and its negative correlation with MDA content. Conversely, the upregulated *Bacteroidales_bacterium_Bact_22* in the CON group showed negative correlations with SOD, CAT, and GSH-Px activities and a positive correlation with MDA content. The findings suggest that dietary EA may enhance Kazakh sheep's antioxidant capacity, partly influenced by rumen microorganisms like *Bacteroidota, Bacteroidales_bacterium_Bact_22*, and *uncultured_rumen_bacterium*.

## 5 Conclusions

In conclusion, the present study demonstrated that the dietary supplementation of 30 mg/kg BW (sheep/day) EA for 5-month-old Kazakh sheep improves the dry matter intake and apparent digestibility of NDF and EE, increases the acetic acid and propionic acid contents in the rumen fluid, regulates the ruminal microbiota, enhances antioxidant capacity, and improves daily weight gain. These findings offer valuable insights into EA supplementation's potential benefits.

## Data availability statement

The original contributions presented in the study are included in the article/supplementary material, further inquiries can be directed to the corresponding author.

## Ethics statement

The animal study was approved by Animal Experiment Ethics Committee of Xin-jiang Agricultural University (permit number: 2020024). The study was conducted in accordance with the local legislation and institutional requirements.

## Author contributions

WZ: Investigation, Visualization, Writing – original draft, Writing – review & editing, Data curation. FR: Investigation, Writing – original draft, Writing – review & editing, Supervision. CZ: Supervision, Writing – original draft, Writing – review & editing. FY: Investigation, Writing – original draft, Writing – review & editing. XuL: Investigation, Writing – original draft, Writing – review & editing. XH: Investigation, Writing – original draft, Writing – review & editing. XiL: Conceptualization, Data curation, Visualization, Writing – original draft, Writing – review & editing. KC: Conceptualization, Funding acquisition, Project administration, Supervision, Writing – original draft, Writing – review & editing.
